# Efficacy and safety of fractional CO_2_ laser therapy combined with triamcinolone acetonide injection for hypertrophic scar: a preliminary systematic review and meta-analysis

**DOI:** 10.3389/fmed.2025.1671191

**Published:** 2026-01-22

**Authors:** Jianfeng Zhang, Mengke Wu, Cong Liu, Xiaochen Zhu, Qin Guo, Cheng Tan

**Affiliations:** Department of Dermatology, Affiliated Hospital of Nanjing University of Chinese Medicine, Jiangsu Province Hospital of Chinese Medicine, Nanjing, China

**Keywords:** fractional CO₂ laser, hypertrophic scar, meta-analysis, randomized controlled trials, triamcinolone acetonide

## Abstract

**Background:**

This study aims to systematically evaluate the efficacy and safety of fractional CO_2_ laser therapy combined with triamcinolone acetonide (TA) injection for hypertrophic scar (HS).

**Materials and methods:**

Randomized controlled trials (RCTs) investigating the combination therapy of fractional CO₂ laser and TA injection for HS were identified through systematic searches of PubMed, Embase, Cochrane Library, Web of Science, China National Knowledge Infrastructure, Wanfang, Sinomed, and VIP databases from inception to December 2024. The risk of bias was assessed using the Cochrane Collaboration’s Risk of Bias tool. Meta-analysis was performed with RevMan (version 5.3), while sensitivity analysis and publication bias assessment were conducted using Stata (version 14.0). The quality of evidence for outcomes was evaluated with the Grades of Recommendations, Assessment, Development, and Evaluation (GRADE) assessment.

**Results:**

Nineteen studies involving 1,775 patients were included in this meta-analysis. Compared with TA injection alone, the pooled results showed that fractional CO₂ laser combined with TA injection significantly reduced Vancouver Scar Scale scores [MD: −2.52, 95% CI: −3.07 to −1.98, *p* < 0.00001], pruritus scores [MD: −0.86, 95% CI: −0.94 to −0.78, *p* < 0.00001], pain scores [MD: −1.04, 95% CI: −1.38 to −0.71, *p* < 0.00001], scar thickness [SMD: −2.36, 95% CI: −3.12 to −1.61, *p* < 0.00001], serum TGF-β1 level [SMD: −2.09, 95% CI: −2.71 to −1.46, *p* < 0.00001], serum VEGF level [SMD: −2.03, 95% CI: −2.90 to −1.17, *p* < 0.00001], serum EGF level [MD: −17.38, 95% CI: −20.94 to −13.82, *p* < 0.00001], and serum TNF-*α* level [SMD: −1.81, 95% CI: −2.77 to −0.85, *p* = 0.0002]. Regarding safety, the combination of fractional CO₂ laser and TA injection reduced the incidence of skin atrophy [RR: 0.52, 95% CI: 0.34 to 0.80, *p* = 0.003] compared to TA injection alone. There was no significant difference between the two groups in the incidence of adverse events, including folliculitis [RR: 0.64, 95% CI: 0.30 to 1.35, *p* = 0.24], erythematous edema [RR: 1.21, 95% CI: 0.68 to 2.16, *p* = 0.52], skin allergies [RR: 0.38, 95% CI: 0.14 to 1.05, *p* = 0.06], pigmentation [RR: 1.12, 95% CI: 0.58 to 2.16, *p* = 0.73], ulcers [RR: 0.35, 95% CI: 0.11 to 1.16, *p* = 0.09], infections [RR: 1.45, 95% CI: 0.29 to 7.17, *p* = 0.65], and blisters [RR: 0.33, 95% CI: 0.01 to 7.88, *p* = 0.50].

**Conclusion:**

Preliminary evidence suggests that fractional CO₂ laser combined with TA injection is an effective treatment for HS. However, due to methodological limitations in the included studies, large-scale, rigorously designed RCTs are required to validate these findings. Additionally, all 19 RCTs were conducted in China with Chinese participants; therefore, the current evidence is limited to this population and requires validation in other ethnic groups.

**Systematic Review Registration:**

https://www.crd.york.ac.uk/PROSPERO/view/CRD42025630116, identifier PROSPERO (CRD4202563011).

## Introduction

Hypertrophic scar (HS) is a pathological condition characterized by the continuous proliferation of scar tissue following the local epithelization of wounds and is a common clinical outcome of excessive tissue repair after burns, injuries, or other types of skin lesions (1). It is estimated that approximately 10–20% of patients may develop hypertrophic scars following an injury, with a higher prevalence observed particularly among younger individuals and women (2). HS not only affects appearance but also cause itching, pain, and even local deformities or dysfunction, significantly impacting patients’ quality of life (3).

The treatment of hypertrophic scars mainly falls into two categories: surgical and non-surgical. Surgical excision alone has a high recurrence rate. Non-surgical options include corticosteroid injections, radiation therapy, laser treatments, and others (4). Finding safe and effective treatments is crucial for managing hypertrophic scars. Fractional CO_2_ laser therapy is a widely used clinical intervention for hypertrophic scars. By generating controlled microthermal injuries in a grid-like pattern, this technique promotes scar tissue vaporization and stimulates cutaneous wound repair mechanisms, ultimately suppressing excessive scar proliferation (5). Its clinical benefits include high specificity, minimal invasiveness, and rapid postoperative recovery (6). Glucocorticoids, particularly triamcinolone acetonide, are a primary treatment for hypertrophic scars. Their mechanism of action involves inhibiting fibroblast proliferation, suppressing collagen synthesis, and promoting collagen degradation, thereby significantly improving scar morphology and texture (7). However, the efficacy of the aforementioned monotherapies often fails to meet clinical expectations, prompting researchers to explore novel therapeutic strategies.

In recent years, numerous clinical studies have explored the use of fractional CO_2_ laser combined with triamcinolone acetonide injection for treating hypertrophic scars. However, the efficacy and safety of this approach have not yet been systematically evaluated.

This study assesses the effectiveness and safety of combining fractional CO_2_ laser with triamcinolone acetonide injection for hypertrophic scar treatment, offering evidence-based insights for clinical practice.

## Methods

This meta-analysis was conducted in accordance with the Preferred Reporting Items for Systematic Reviews and Meta-Analysis (PRISMA) guidelines (8) (Supplementary material S1) and was prospectively registered on the PROSPERO platform (*CRD42025630116*).

### Search strategy

A comprehensive search was conducted across eight databases, including PubMed, Embase, the Cochrane Library, Web of Science, China National Knowledge Infrastructure (CNKI), Wanfang, Sinomed, and VIP, covering all available records from their inception through December 2024, to identify relevant studies. The search terms were presented as: “Hypertrophic Scar,” “Hypertrophic Scarring,” “Scar, Hypertrophic,” “Keloid,” “Keloid Scar,” “Cicatrix, Hypertrophic,” “Scar Tissue,” “Scar Revision,” “Carbon dioxide laser,” “CO_2_ laser,” “Fractional laser,” “Fractional carbon dioxide laser,” “Fractional CO_2_ laser,” “Triamcinolone Acetonide,” “Acetonide, Triamcinolone.” The search strategy was customized to suit the specific requirements of each database. No restrictions were imposed regarding language or publication status. Additionally, the reference lists of the included studies were manually reviewed to identify potentially eligible research. The search strategy of PubMed is taken as an example, as shown in Supplementary material S2.

### Inclusion and exclusion criteria

The meta-analysis included trials meeting these criteria: (1) randomized controlled trials (RCTs). (2) participants with a confirmed diagnosis of hypertrophic scars (irrespective of age, gender, or ethnicity) were enrolled, whereas patients with keloids or mixed pathological scars were excluded. (3) experimental intervention involving fractional CO_2_ laser combined with triamcinolone acetonide injection (laser types, settings, and regimens unrestricted), and the control intervention using the same triamcinolone acetonide regimen as the experimental group, with consistent baseline treatments in both groups. (4) primary outcome was assessed using the Vancouver Scar Scale (VSS) score (9), with secondary outcomes including the score of pruritus, the score of pain, scar thickness, serum levels of TGF-β1, VEGF, EGF, TNF-*α*, and adverse events.

Studies that met the following criteria were excluded: (1) non-randomized controlled trials (non-RCTs) such as editorials, case reports, review articles, conference abstracts, and animal studies. (2) publications lacking sufficient data or with inaccessible full-text content. (3) duplicate publications.

### Data extraction

Two independent researchers performed the data extraction process, with cross-verification conducted simultaneously. Any discrepancies between the researchers were resolved through consensus-based discussions. In cases where the published literature contained incomplete data, attempts were made to contact the corresponding authors via email or telephone. Studies with unavailable or inaccessible data were subsequently excluded from the analysis. The data extraction encompassed multiple categories, including: (1) general study characteristics (first author’s name, country of origin, and publication year). (2) baseline demographic information (participant age, gender distribution, and sample size). (3) intervention protocols for both experimental and control groups. (4) outcome measures, and (5) specific parameters of laser treatment protocols.

### Assessment of study quality

Two researchers independently assessed the risk of bias in the trials following the guidelines outlined in the Cochrane Handbook for Systematic Reviews of Interventions (10). The evaluation was conducted using seven specific criteria: random sequence generation, allocation concealment, blinding of participants and personnel, blinding of outcome assessment, incomplete outcome data, selective reporting, and other potential sources of bias. Each criterion was categorized as having a low, unclear, or high risk of bias. Any disagreements in the assessments were resolved by a third-party.

### Level of evidence

Using the GRADE criteria, we evaluated the evidence quality of outcomes via the GRADEpro GDT platform^1^ (11). Evidence levels were categorized as very low (+), low (++), moderate (+++), or high (++++) based on factors such as bias risk, inconsistency, indirectness, imprecision, and other considerations.

### Statistical analysis

We conducted a meta-analysis using Revman 5.3. Dichotomous outcomes were assessed with risk ratios (RR) and 95% confidence intervals (CI). For continuous outcomes, we calculated either the mean difference (MD) or the standardized mean difference (SMD), each with a 95% CI. The choice between MD and SMD depended on whether the outcomes were measured on the same or different scales. Statistical significance was set at *p* < 0.05. Heterogeneity was assessed via the χ^2^ and I^2^ tests, with a random-effects model applied if *p* ≤ 0.1 and I^2^ ≥ 50%, and a fixed-effects model if *p* > 0.1 and I^2^ < 50%. Subgroup analyses by intervention and comparison were performed by total number of laser sessions and treatment interval. Other parameters (fluence, pulse duration, TA dose, follow-up time) could not be examined due to incomplete reporting. To assess the robustness of the findings, sensitivity analysis was conducted using Stata 14.0. Funnel plots, Begg’s test (12), and Egger’s test (13) were used to assess publication bias when more than 10 studies were included.

## Results

### Study selection

**Figure 1:**
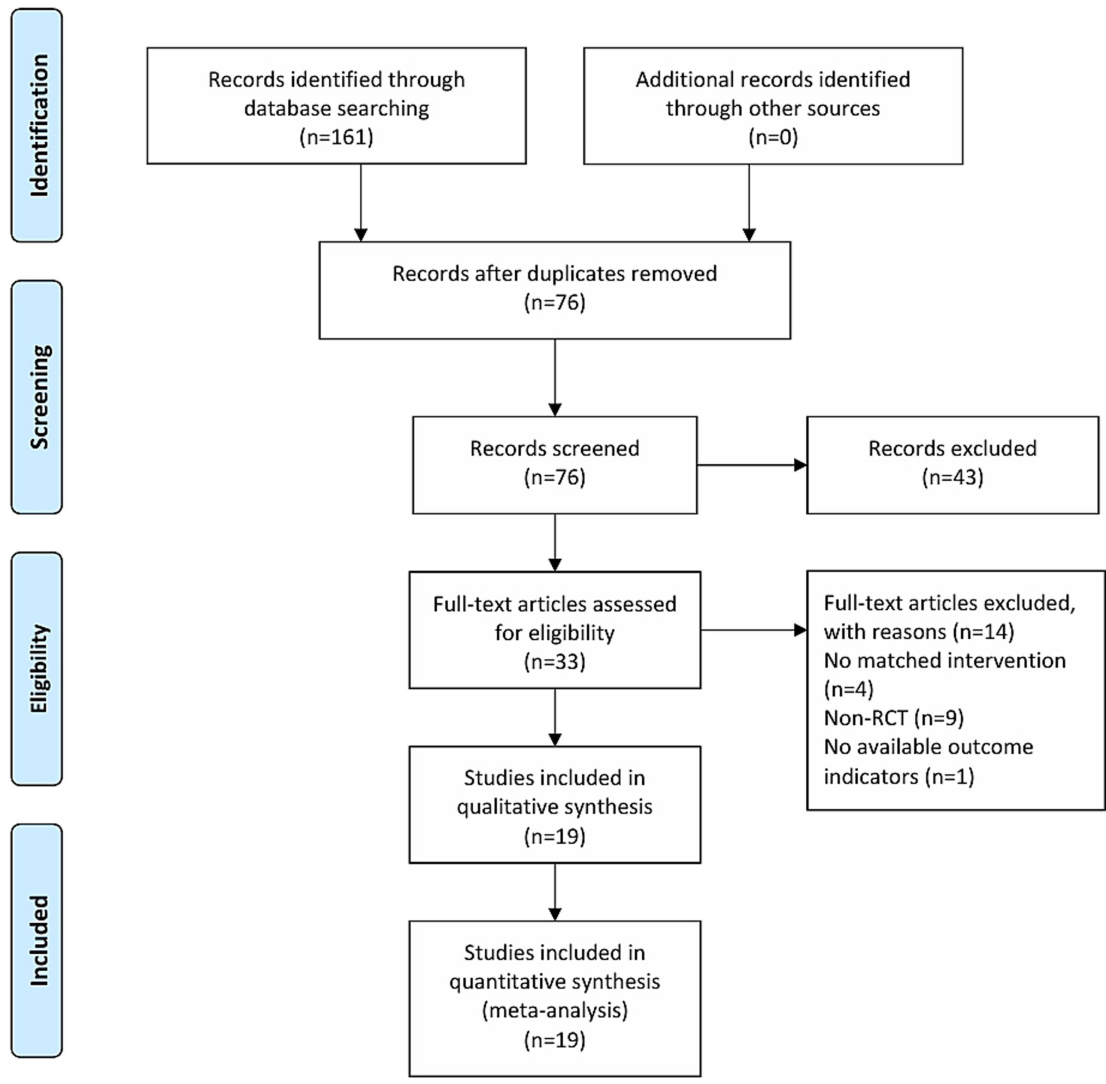
The flowchart of study selection.

A total of 161 citations were identified through the initial search strategy. Following the removal of 85 duplicates and title/abstract screening excluding 43 irrelevant studies, 33 records underwent full-text assessment, of which 14 were excluded. Ultimately, 19 studies (14–32) met the inclusion criteria and were included in the analysis (Figure 1).

### Study characteristics

Nineteen studies involving a total of 1775 patients (889 in the experimental group and 886 in the control group) were published between 2016 and 2024. The sample size ranged from 19 to 112. All studies were conducted in China, and all participants were Chinese. The characteristics of the included studies are shown in Table 1. In the experimental group, fractional CO₂ laser was applied in all cases, and the laser treatment settings are summarized in Table 2.

**Table 1:** Features of the involved studies.

Study and year	Size (T/C)	Sex (Male/Female)	Age (years)	Baseline comparable	T groups interventions	C groups interventions	Treatment course	Outcomes
Zheng JX 2020 (14)	29/29	T:11/18 C:10/19	T:19–58 C:18–57	Yes	Fractional CO_2_ laser + CG	TA injection + mupirocin ointment	3 m	②③⑤⑥⑨
Ding F 2018 (15)	52/52	T:22/30 C:20/32	T:21–62 C:20–61	Yes	Fractional CO_2_ laser + CG	TA injection	20w	⑨
Kou CC 2022 (16)	20/19	T:9/11 C:7/12	T: 32.74 ± 3.62 C: 32.39 ± 3.39	Yes	Fractional CO_2_ laser + CG	TA injection	2 m	①④
Li SL 2019 (17)	35/35	T:17/18 C:19/16	T:22–53 C:21–51	Yes	Fractional CO_2_ laser + CG	TA injection	3 m	①④⑤
Li ZZ 2024 (18)	39/39	T:27/12 C:25/14	T:19–47 C:19–45	Yes	Fractional CO_2_ laser + CG	TA injection	12w	①④⑨
Liu YL 2021 (19)	40/40	T:26/14 C:28/12	T:18–60 C:18–59	Yes	Fractional CO_2_ laser + CG	TA injection+ halometasone triclosan cream	24w	①②③⑨
Wang FY 2024 (20)	102/102	T:58/44 C:56/46	T:32.45 ± 6.89 C:33.14 ± 7.21	Yes	Fractional CO_2_ laser + CG	TA injection	6 m	①②⑤⑥⑦⑧⑨
Wang M 2024 (21)	58/58	T:30/28 C:31/27	T:21–57 T:21–57	Yes	Fractional CO_2_ laser + CG	TA injection + hyaluronic acid	24w	①②③⑨
Wang Q 2023 (22)	36/36	T:17/19 C:15/21	T:34.72 ± 5.28 C:35.13 ± 5.72	Yes	Fractional CO_2_ laser + CG	TA injection	12w	①④⑤⑥⑧⑨
Wang ZC 2024 (23)	50/50	T:22/28 C:25/25	T:20–57 C:20–56	Yes	Fractional CO_2_ laser + CG	TA injection	3 m	②③⑤⑥
Wu XR 2019 (24)	112/108	T:54/58 C:55/53	T:23–45 C:21–46	Yes	Fractional CO_2_ laser + CG	TA injection	12w	①⑨
Yin ZY 2017 (25)	30/29	T:11/19 C:12/17	T: 18–62 C: 16–60	Yes	Fractional CO_2_ laser + CG	TA injection	3 m	①
Zhang PL 2019 (26)	36/34	T:23/13 C:20/14	T:20–40 C:21–51	Yes	Fractional CO_2_ laser + CG	TA injection	24w	①④⑨
Zhang XZ 2019 (27)	28/28	T:11/17 C:10/18	T:19–48 C:18–47	Yes	Fractional CO_2_ laser + CG	TA injection	24w	①④⑨
Zheng ML 2016 (28)	30/30	T:11/19 C:14/16	T:15–63 C:15–63	Yes	Fractional CO_2_ laser + CG	TA injection	24w	①⑨
Zheng YH 2024 (29)	34/34	T:17/17 C:16/18	T:40.25 ± 1.85 C:40.19 ± 1.74	Yes	Fractional CO_2_ laser + CG	TA injection	6w	①④⑤⑥⑦⑧⑨
Zhou YP 2021 (30)	82/87	T:48/34 C:47/40	T:18–50 T:18–60	Yes	Fractional CO_2_ laser + CG	TA injection	1w	①⑨
Zhu TT 2022 (31)	31/31	T:19/12 C:18/13	T:38.85 ± 2.48 C:38.51 ± 2.51	Yes	Fractional CO_2_ laser + CG	TA injection	5 m	①③⑨
Zhuang MS 2017 (32)	45/45	T:22/23 C:23/22	T:21–39 C:22–40	Yes	Fractional CO_2_ laser + CG	TA injection	24w	①④⑨

T: treatment; C: control; CG: control group; w: week; m: month; TA: triamcinolone acetonide; ①: Vancouver Scar Scale (VSS) scores; ②: pruritus scores; ③: pain scores; ④: scar thickness;⑤: the level of serum TGF-β1; ⑥: the level of serum VEGF; ⑦: the level of serum EGF; ⑧: The level of serum TNF-α; ⑨: adverse events.

**Table 2:** Laser treatment settings.

Study and Year	Type of laser	Equipment origin	Fluence (J/cm^2^)	Pulse duration (msec)	Spot size (mm)	Frequency (Hz)	Temperature (°C)	Passes	Total sessions	Treatment interval
Zheng JX 2020 (14)	Fractional CO_2_ laser	China	NR	2–3	NR	NR	NR	NR	3	1 m
Ding F 2018 (15)	Fractional CO_2_ laser	China	5–100	NR	NR	NR	NR	NR	5	4w
Kou CC 2022 (16)	Fractional CO_2_ laser	NR	40–70	NR	NR	300	NR	NR	4	2w
Li SL 2019 (17)	Fractional CO_2_ laser	China	NR	NR	NR	NR	NR	NR	6	0.5 m
Li ZZ 2024 (18)	Fractional CO_2_ laser	China	70–120	NR	5	NR	NR	NR	3	4w
Liu YL 2021 (19)	Fractional CO_2_ laser	China	5–100	NR	NR	NR	NR	NR	6	4w
Wang FY 2024 (20)	Fractional CO_2_ laser	China	40–50	NR	NR	NR	NR	NR	6	1 m
Wang M 2024 (21)	Fractional CO_2_ laser	China	5–100	NR	NR	NR	NR	NR	6	4w
Wang Q 2023 (22)	Fractional CO_2_ laser	China	7–16.9	NR	NR	10–60	NR	NR	3	4w
Wang ZC 2024 (23)	Fractional CO_2_ laser	China	NR	NR	NR	NR	NR	NR	3	1 m
Wu XR 2019 (24)	Fractional CO_2_ laser	China	40–70	NR	NR	300	NR	NR	3	4w
Yin ZY 2017 (25)	Fractional CO_2_ laser	China	NR	NR	NR	NR	NR	NR	12	1w
Zhang PL 2019 (26)	Fractional CO_2_ laser	NR	12	NR	NR	300	NR	NR	6	4w
Zhang XZ 2019 (27)	Fractional CO_2_ laser	South Korea	15	NR	100	300	NR	NR	6	4w
Zheng ML 2016 (28)	Fractional CO_2_ laser	China	NR	NR	NR	NR	NR	NR	6	4w
Zheng YH 2024 (29)	Fractional CO_2_ laser	American	100–1,000	NR	5	NR	NR	NR	6	1w
Zhou YP 2021 (30)	Fractional CO_2_ laser	China	NR	1–3	NR	NR	NR	NR	1	1w
Zhu TT 2022 (31)	Fractional CO_2_ laser	China	NR	NR	NR	300	NR	NR	2	2 m
Zhuang MS 2017 (32)	Fractional CO_2_ laser	NR	15	NR	100	300	NR	NR	6	4w

NR, not reported; w, week; m, month.

### Assessment of risk of bias

**Figure 2:**
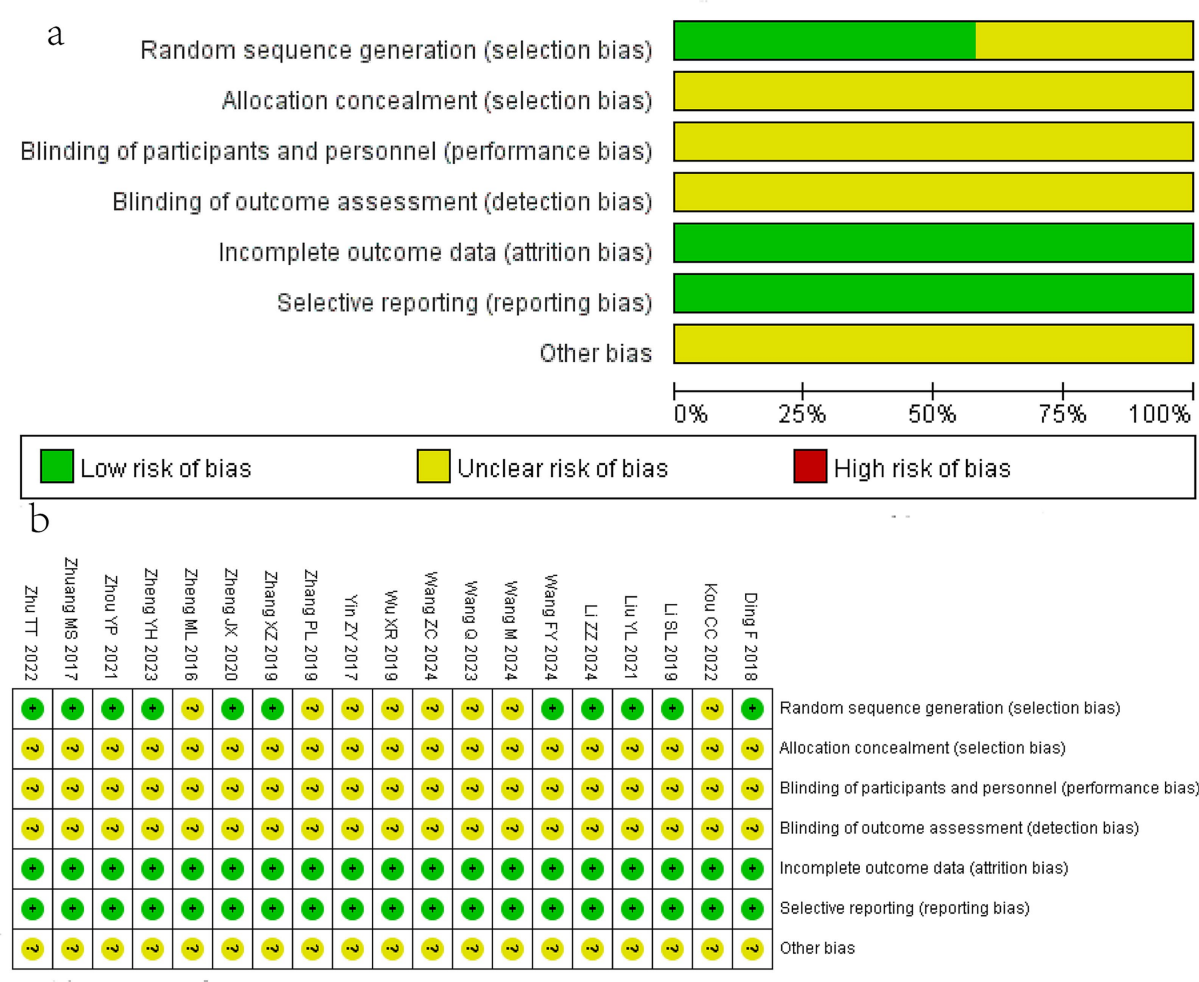
Bias risk assessment of included studies. **(a)** Risk of bias of each study; **(b)** Summary of risk of bias.

Eleven studies (14, 15, 17–20, 27, 29–32) described random sequence generation using the random number table method. None of the studies mentioned allocation concealment, blinding of participants and personnel, or evaluator blinding. Additionally, no included studies had incomplete outcome data or selective reporting. Other potential biases remained unclear due to insufficient information. The details of the risk of bias assessment are presented in Figure 2.

### Meta-analysis results

#### Vancouver Scar Scale scores

**Figure 3:**
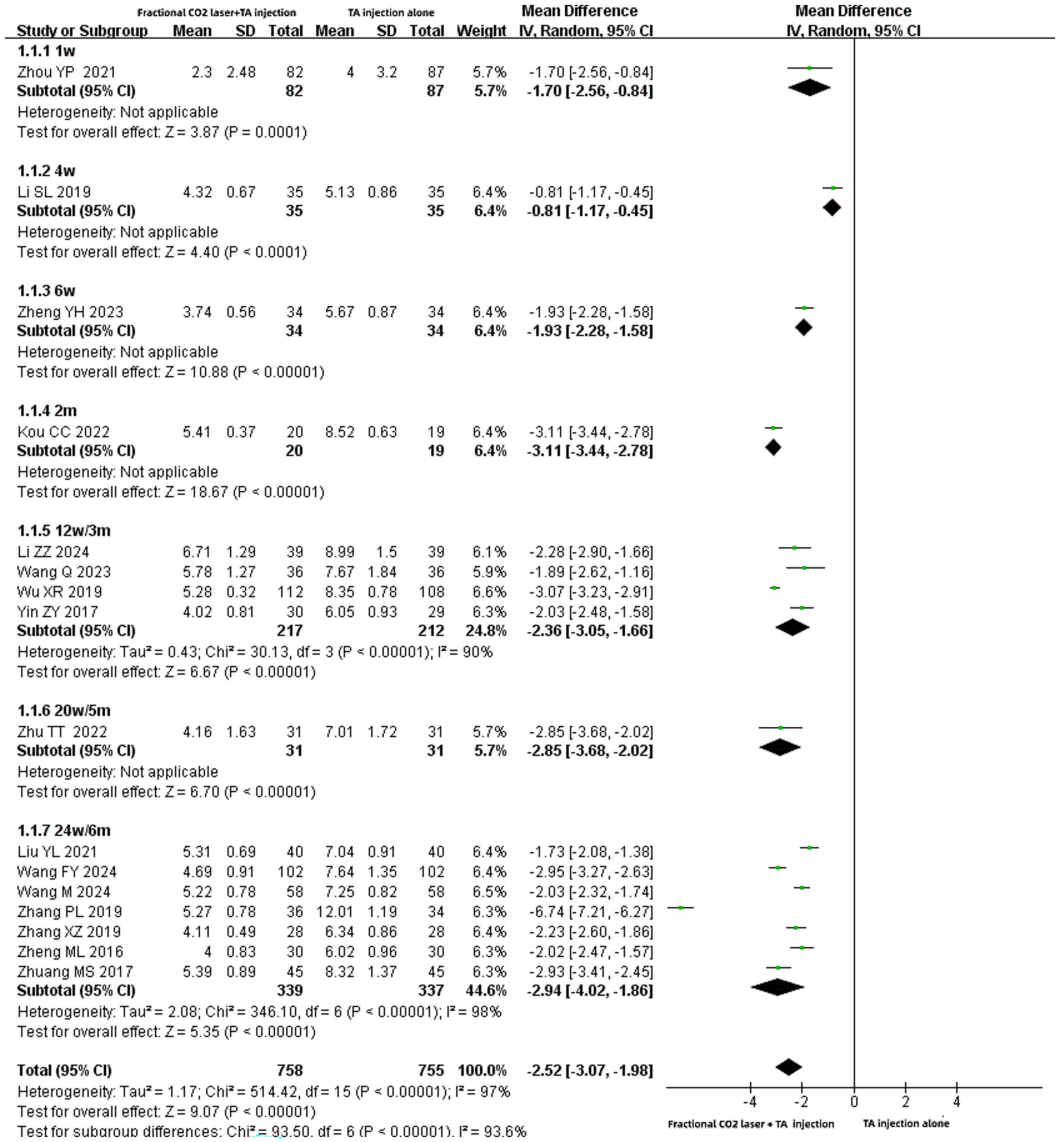
Forest plot for VSS scores.

Sixteen studies (16–25, 27–32) reported VSS score with a total of 1,513 patients (758 patients in the experimental group and 755 patients in the control group). Due to significant data heterogeneity (I^2^ = 97%, *p* < 0.00001), a random-effects model was applied. The meta-analysis results suggested that the combination of fractional CO₂ laser and TA injection significantly reduced the VSS scores compared with triamcinolone acetonide injection alone [MD: −2.52, 95% CI: −3.07 to −1.98, *p* < 0.00001] (Figure 3). Subgroup analysis was performed based on the treatment course of fractional CO₂ laser. The VSS scores in the group receiving fractional CO₂ laser (1-week course) plus TA injection were significantly lower than in the TA injection-only group [MD: −1.70, 95% CI: −2.56 to −0.84, *p* = 0.0001]. Similar results were observed for the following combination therapy courses: 4 weeks [MD: −0.81, 95% CI: −1.17 to −0.45, *p* < 0.0001], 6 weeks[MD: −1.93, 95% CI: −2.28 to −1.58, *p* < 0.00001], 2 months [MD: −3.11, 95% CI: −3.44 to −2.78, *p* < 0.00001], 12 weeks (or 3 months) [MD: −2.36, 95% CI: −3.05 to −1.66, *p* < 0.00001], 20 weeks (or 5 months) [MD: −2.85, 95% CI: −3.68 to −2.02, *p* < 0.00001], 24 weeks (or 6 months) [MD: −2.94, 95% CI: −4.02 to −1.86, *p* < 0.00001].

#### Pruritus scores

**Figure 4:**
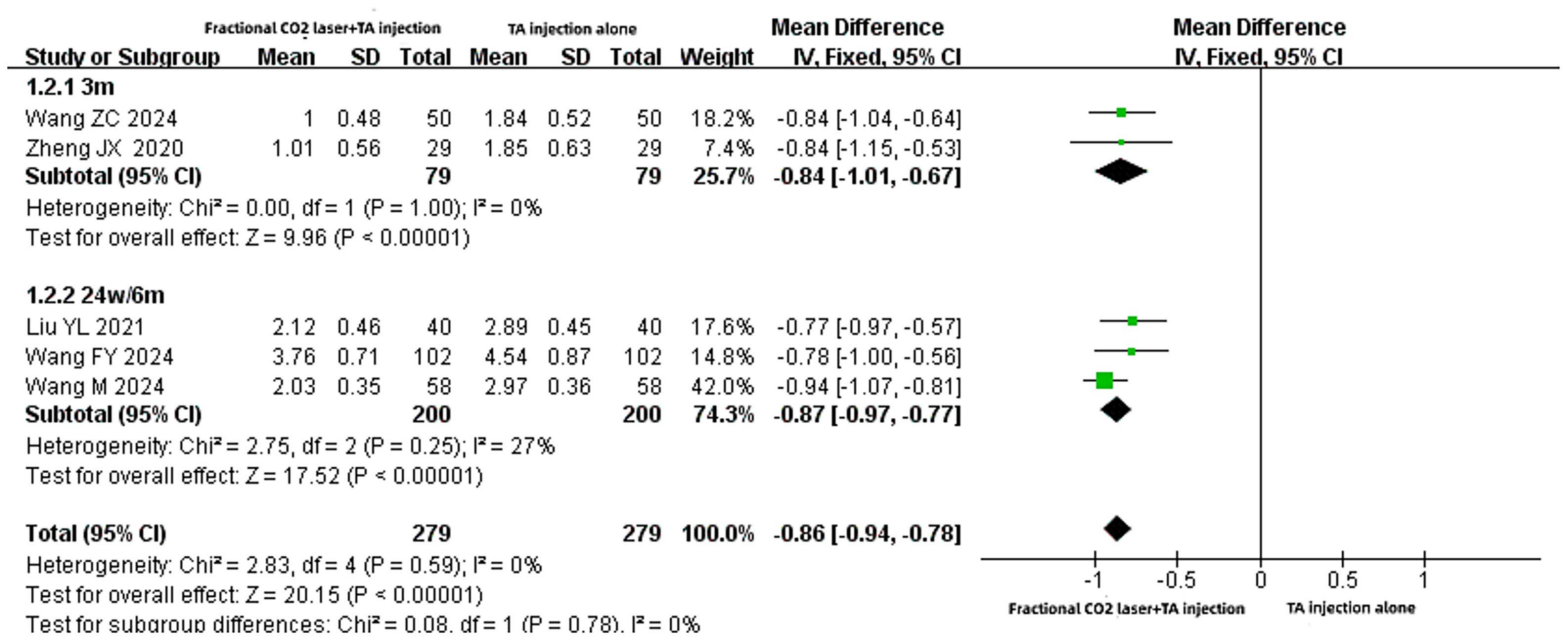
Forest plot for pruritus scores.

A total of five studies (14, 19–21, 23) involving 558 patients reported pruritus scores. Meta-analysis demonstrated that the combination of fractional CO₂ and TA injection significantly reduced pruritus scores compared to TA monotherapy alone [MD: −0.86, 95% CI: −0.94 to −0.78, *p* < 0.00001] (Figure 4). Subgroup analyses confirmed this benefit across different treatment durations (3 months or 24 weeks/6 months) [MD: −0.84, 95% CI: −1.01 to −0.67, *p* < 0.00001; MD: −0.87, 95% CI: −0.97 to −0.77, *p* < 0.00001].

#### Pain scores

**Figure 5:**
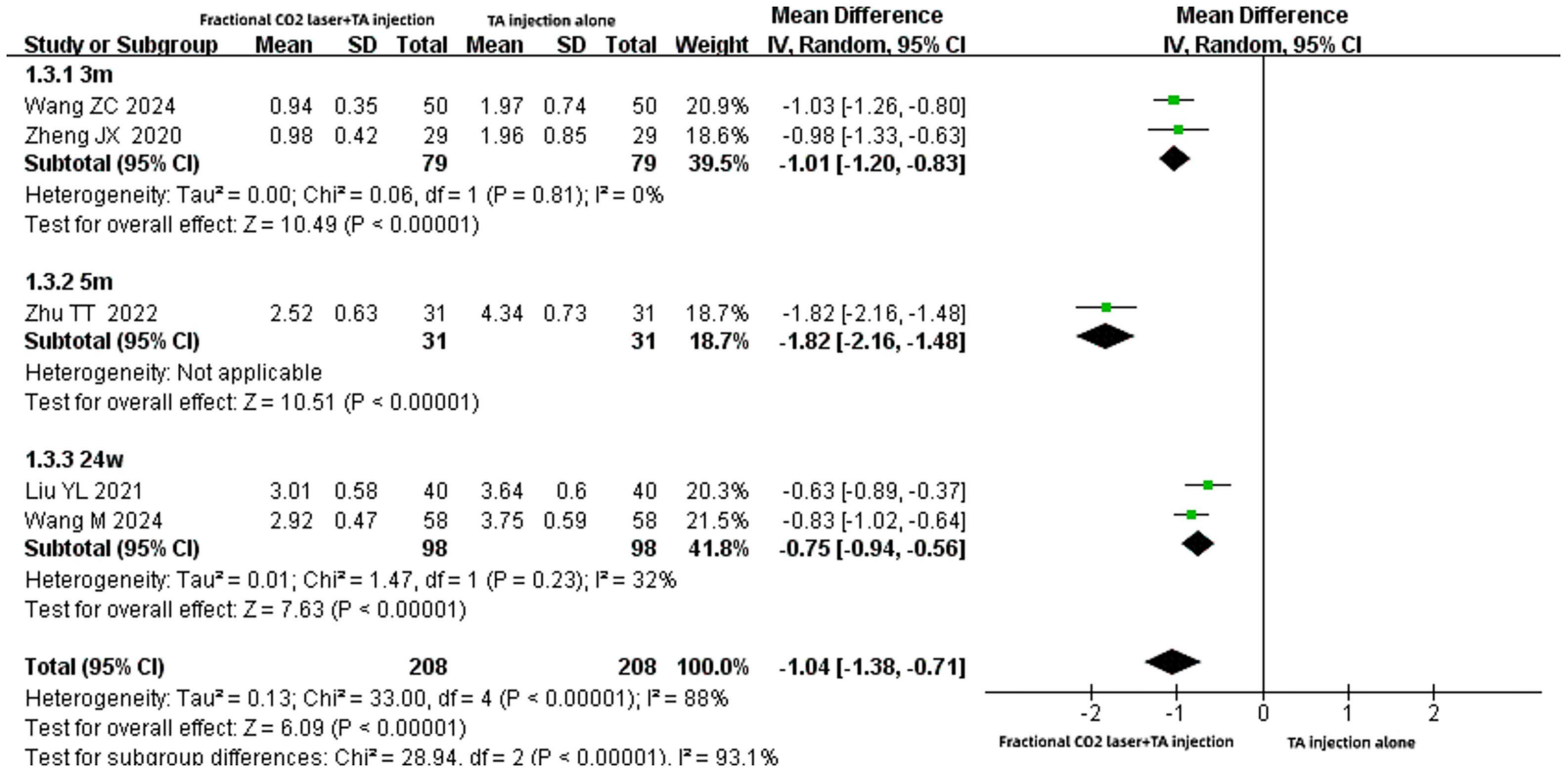
Forest plot for pain scores.

Five studies (14, 19, 21, 23, 31) involving 416 patients reported pain score outcomes. Meta-analysis suggested that the combination of fractional CO₂ laser with TA injection significantly reduced pain scores compared to TA monotherapy [MD: −1.04, 95% CI: −1.38 to −0.71, *p* < 0.00001] (Figure 5). Subgroup analyses consistently showed superior pain reduction with combination therapy across all treatment durations examined 3 months [MD: −1.01, 95% CI: −1.20 to −0.83, *p* < 0.00001], 5 months [MD: −1.82, 95% CI: −2.16 to −1.48, *p* < 0.00001], or 24 weeks [MD: −0.75, 95% CI: −0.94 to −0.56, *p* < 0.00001].

### Scar thickness

**Figure 6:**
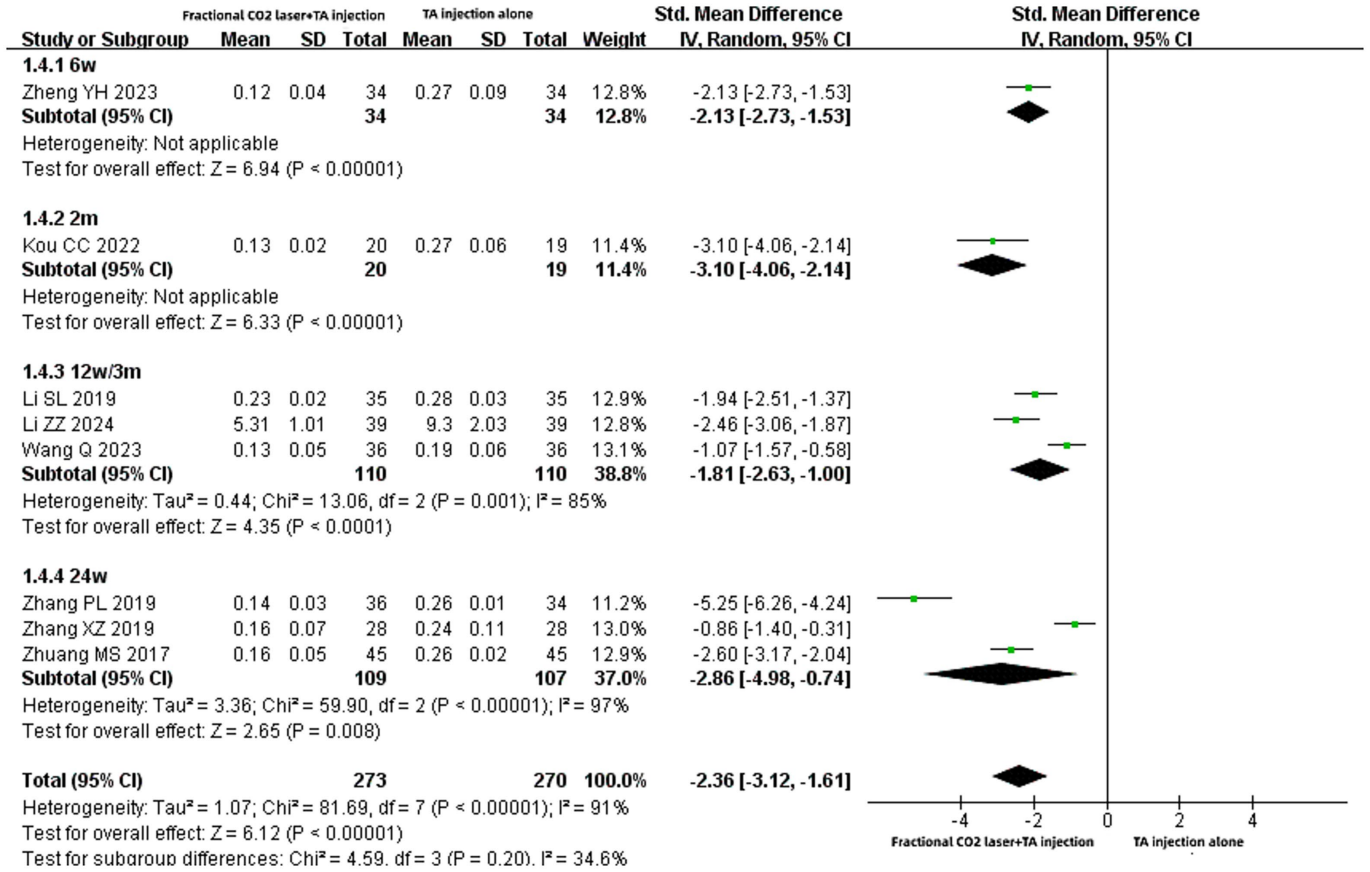
Forest plot for scar thickness.

As eight records (16–18, 22, 26, 27, 29, 32) reported scar thickness with different units (e.g., mm, cm), SMD was applied to evaluate the effects. The pooled results indicated that fractional CO₂ combined with TA injection significantly reduced scar thickness compared to TA injection alone [SMD: −2.36, 95% CI: −3.12 to −1.61, *p* < 0.00001] (Figure 6). Subgroup analyses demonstrated that the fractional CO₂ plus TA injection group achieved greater scar thickness reduction than the TA injection alone group, regardless of treatment duration of 6 weeks[SMD: −2.13, 95% CI: −2.73 to −1.53, *p* < 0.00001], 2 months[SMD: −3.10, 95% CI: −4.06 to −2.14, *p* < 0.00001], 12 weeks (3 months) [SMD: −1.81, 95% CI: −2.63 to −1.00, *p* < 0.0001], or 24 weeks[SMD: −2.86, 95% CI: −4.98 to −0.74, *p* = 0.008].

### The level of serum TGF-β1

**Figure 7:**
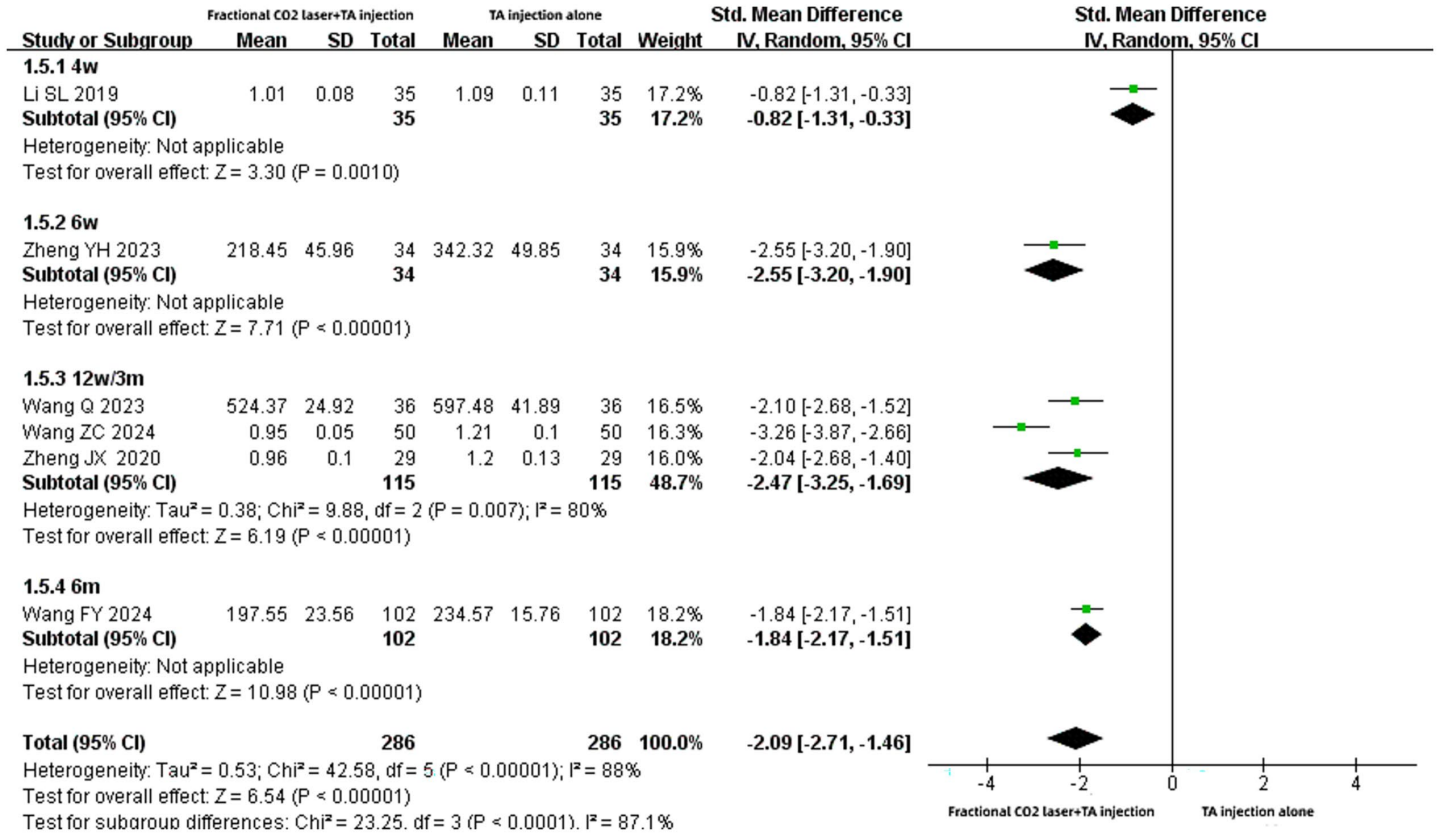
Forest plot for serum TGF-β1 level.

Since six studies (14, 17, 20, 22, 23, 29) reported serum TGF-β1 levels in different units (e.g., μg/L, ng/mL, and ng/L), SMD was applied to assess the effects. The pooled results suggested that the combination of fractional CO₂ and TA injection significantly reduced serum TGF-β1 levels compared with TA injection alone [SMD: −2.09, 95% CI: −2.71 to −1.46, *p* < 0.00001] (Figure 7). Subgroup analyses yielded similar results regardless of treatment duration of 4 weeks [SMD: −0.82, 95% CI: −1.31 to −0.33, *p* = 0.001], 6 weeks [SMD: −2.55, 95% CI: −3.20 to −1.90, *p* < 0.00001], 12 weeks (3 months) [SMD: −2.47, 95% CI: −3.25 to −1.69, *p* < 0.00001], or 6 months[SMD: −1.84, 95% CI: −2.17 to −1.51, *p* < 0.00001].

### The level of serum VEGF

**Figure 8:**
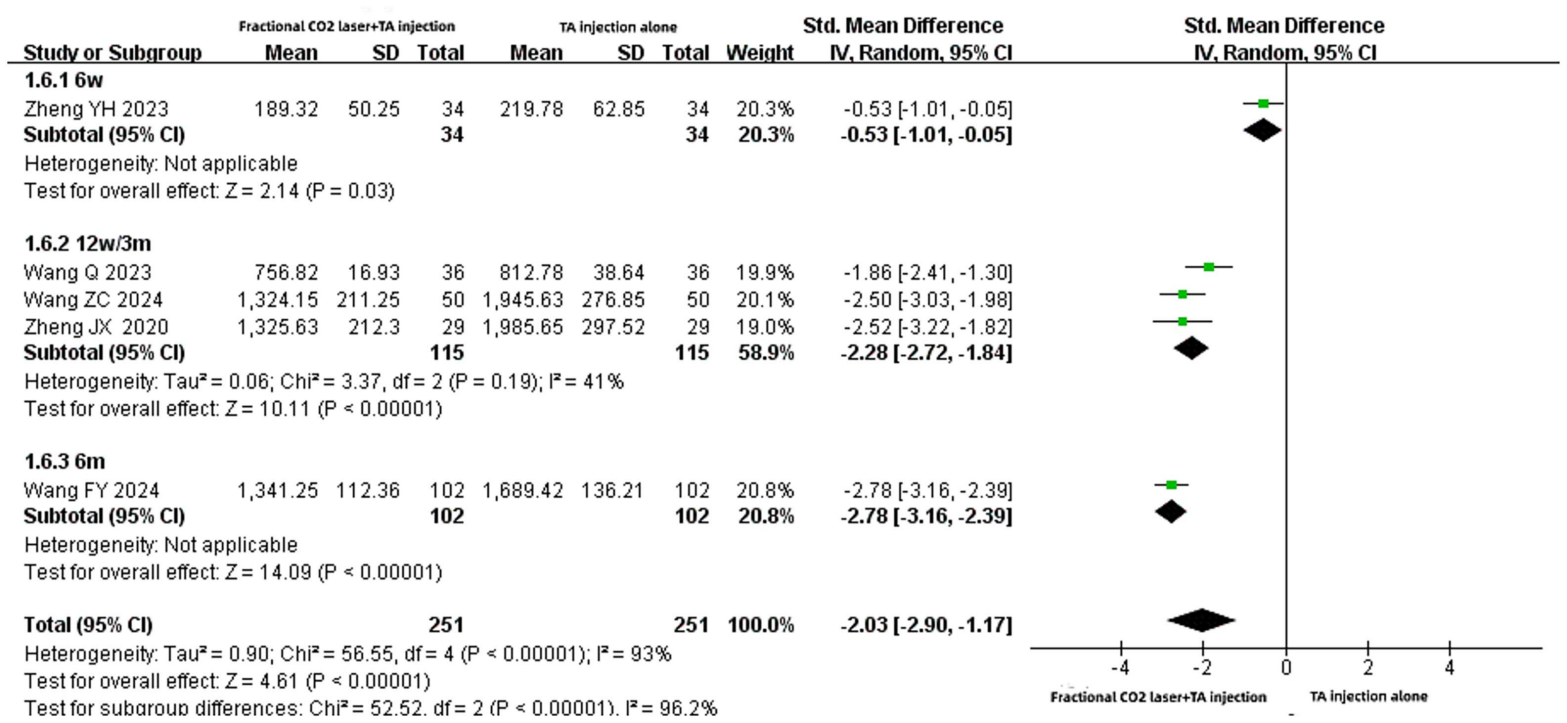
Forest plot for serum VEGF level.

Five records (14, 20, 22, 23, 29) reported serum VEGF level. The unit “ng/L” was used in the two studies, while “ug/L” was used in the other three. The meta-analysis revealed that the serum VEGF level in the fractional CO₂ laser combined with TA injection group was statistically lower than that in the TA injection alone group [SMD: −2.03, 95% CI: −2.90 to −1.17, *p* < 0.00001] (Figure 8). Subgroup analyses showed that this combined therapy significantly decreased serum VEGF levels compared with TA injection alone across different treatment durations: 6 weeks [SMD: −0.53, 95% CI: −1.01 to −0.05, *p* = 0.03], 12 weeks (3 months) [SMD: −2.28, 95% CI: −2.72 to −1.84, *p* < 0.00001], and 6 months [SMD: −2.78, 95% CI: −3.16 to −2.39, *p* < 0.00001].

### The level of serum EGF

**Figure 9:**
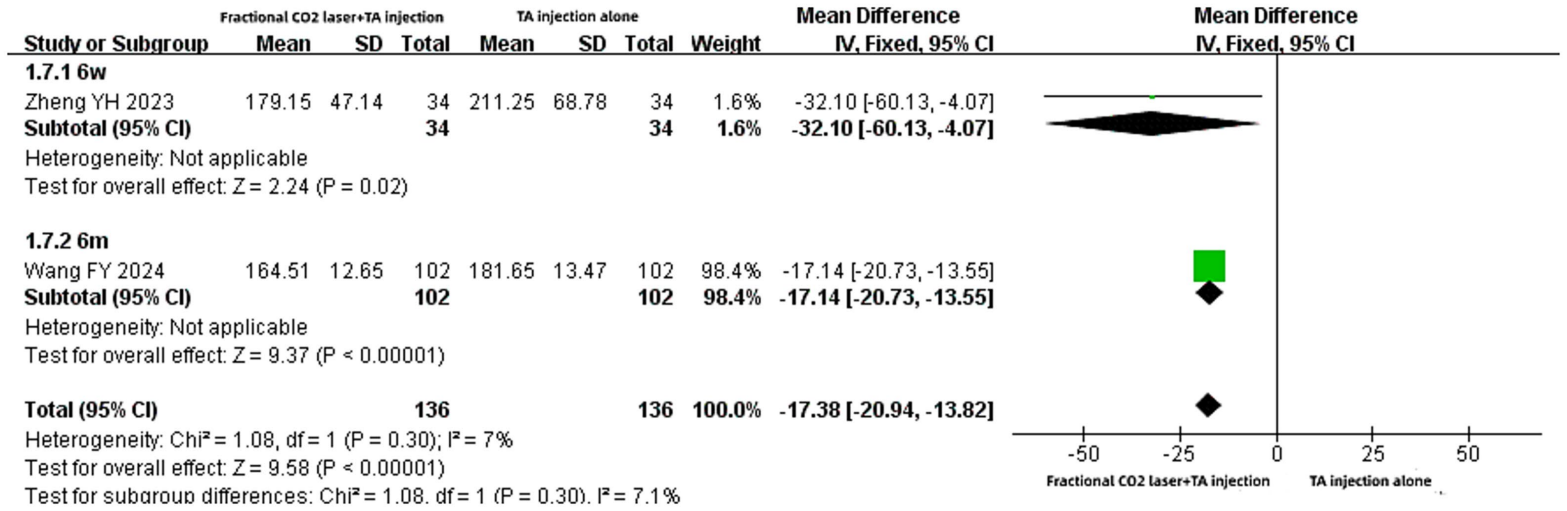
Forest plot for serum EGF level.

Only two studies (20, 29) reported the serum EGF level with the unit “ng/L.” Given the absence of significant heterogeneity (I^2^ = 7%, *p* = 0.30), a fixed-effects model was used. The meta-analysis suggested that combined therapy significantly reduced the serum EGF level compared with TA injection alone [MD: −17.38, 95% CI: −20.94 to −13.82, *p* < 0.00001] (Figure 9). Regardless of whether the treatment course was 6 weeks [MD: −32.10, 95% CI: −60.13 to −4.07, *p* = 0.02] or 6 months [MD: −17.14, 95% CI: −20.73 to −13.55, *p* < 0.00001], subgroup analysis yielded consistent results.

### The level of serum TNF-*α*

**Figure 10:**
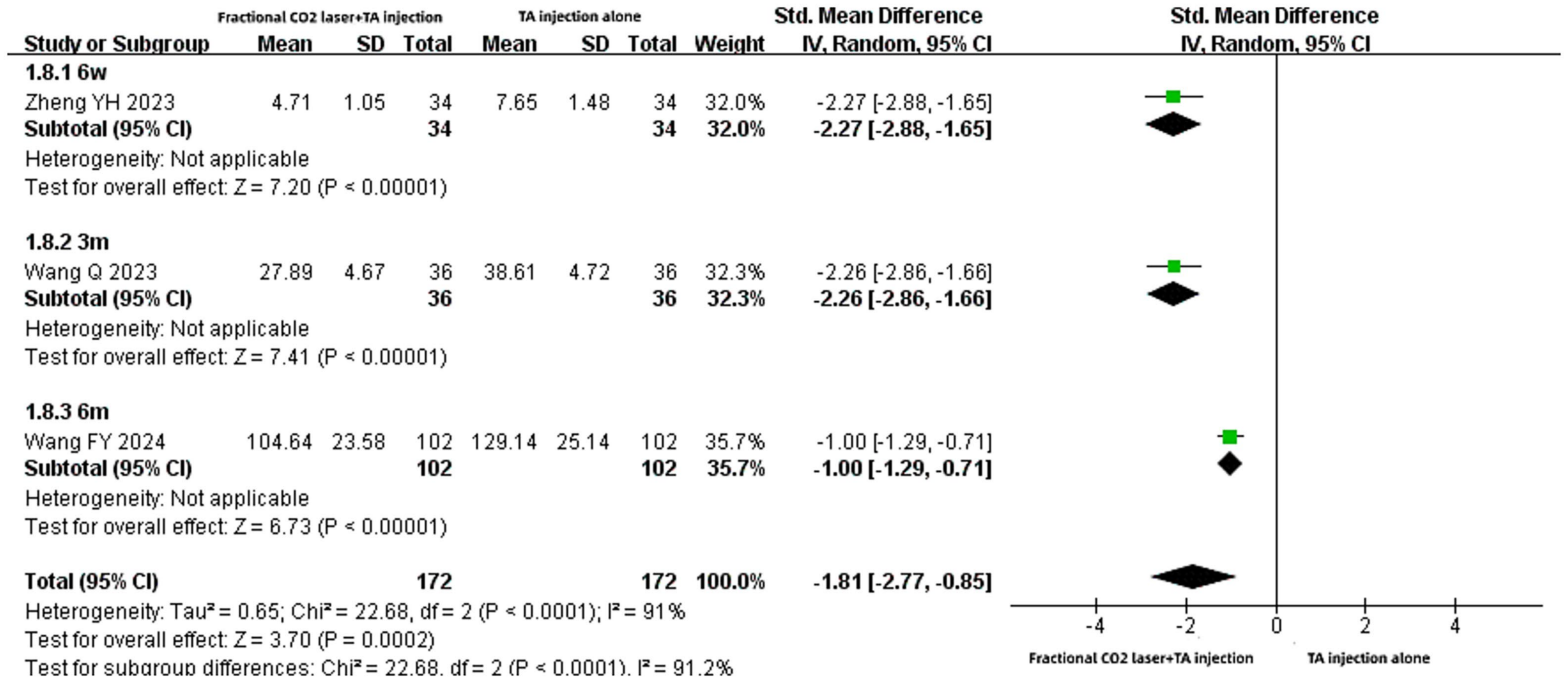
Forest plot for serum TNF-*α* level.

Three studies (20, 22, 29) reported serum TNF-α levels with different units (ng/mL or ng/L). The treatment course involved 6 weeks, 3 months, or 6 months. Regardless of whether pooled results or subgroup analyses were considered, the fractional CO₂ laser combined with TA injection group showed significantly lower serum TNF-α levels than the TA injection-alone group [SMD: −2.27, 95% CI: −2.88 to −1.65, *p* < 0.00001; SMD: −2.26, 95% CI: −2.86 to −1.66, *p* < 0.00001; SMD: −1.00, 95% CI: −1.29 to −0.71, *p* < 0.00001] (Figure 10).

### Adverse events

Fifteen studies (14, 15, 18–22, 24, 26–32) showed adverse events. Only one record (14) failed to specify the number of adverse event cases. In this study, patients in both groups exhibited pain symptoms of varying degrees post-treatment, but these symptoms resolved spontaneously within several hours. In the combined therapy group, some patients developed edema, erythema, punctate bleeding, and a burning sensation following laser treatment; these adverse effects subsided with ice application.

**Figure 11:**
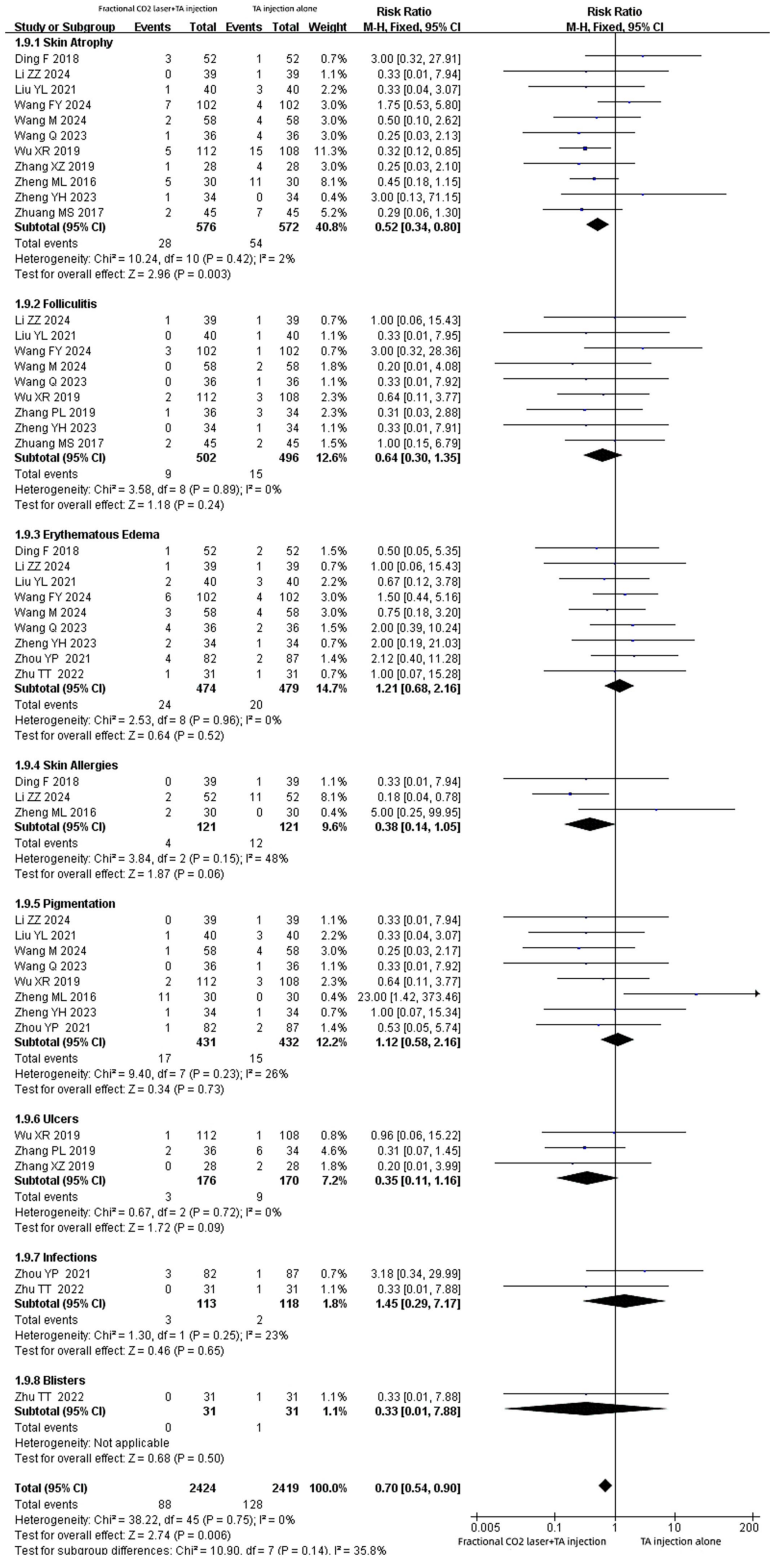
Forest plot for adverse events.

Eight symptoms associated with adverse events-such as skin atrophy, folliculitis, erythematous edema, skin allergies, pigmentation, ulcers, infections, and blisters-were documented in the remaining 14 studies (15, 18–22, 24, 26–32). Subgroup analyses were conducted for different adverse event symptoms (Figure 11). The pooled results indicated that fractional CO₂ laser combined with TA injection had a lower incidence of skin atrophy compared with TA injection alone [RR: 0.52, 95% CI: 0.34 to 0.80, *p* = 0.003]. Nevertheless, no significant difference was observed between the two groups in the incidence of folliculitis [RR: 0.64, 95% CI: 0.30 to 1.35, *p* = 0.24], erythematous edema [RR:1.21, 95% CI: 0.68 to 2.16, *p* = 0.52], skin allergies [RR: 0.38, 95% CI: 0.14 to 1.05, *p* = 0.06], pigmentation[RR:1.12, 95% CI: 0.58 to 2.16, *p* = 0.73], ulcers[RR:0.35, 95% CI: 0.11 to 1.16, *p* = 0.09], infections [RR:1.45, 95% CI: 0.29 to 7.17, *p* = 0.65], or blisters [RR:0.33, 95% CI: 0.01 to 7.88, *p* = 0.50].

### Additional analysis

#### Subgroup analyses

First, a subgroup analysis was conducted based on the total number of laser sessions. The results of this subgroup analysis showed that, compared with the control group treated with TA injection alone, the combined treatment group was superior in improving VSS scores, pruritus scores, pain scores, and scar thickness, as well as in reducing the serum levels of TGF-β1, VEGF, and TNF-*α* (Supplementary File S3). Similar results were obtained from the subgroup analysis based on treatment intervals. The combined treatment group also outperformed the control group in improving VSS scores, pain scores, and scar thickness, and in lowering the serum levels of TGF-β1, VEGF, EGF, and TNF-α (Supplementary File S3).

#### Sensitivity analysis

**Figure 12:**
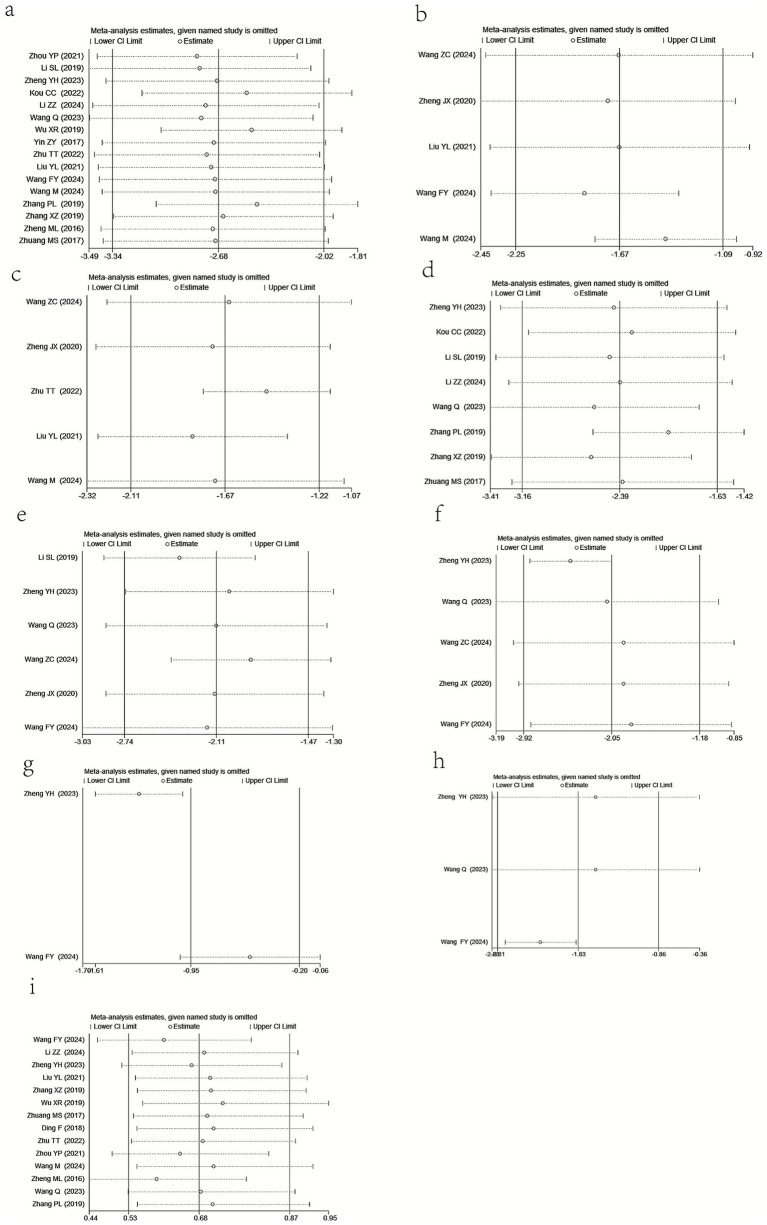
The results of sensitivity analysis: **(a)** VSS scores, **(b)** pruritus scores, **(c)** pain scores, **(d)** scar thickness, **(e)** serum TGF-β1 level, **(f)** serum VEGF level, **(g)** serum EGF level, **(h)** serum TNF-α level, and **(I)** adverse events.

A sensitivity analysis based on the leave-one-out strategy was performed to test the robustness of the pooled results of our meta-analysis. There was no significant change in the effect size and its 95% CIs each time a single study was removed from the pooled analysis (Figure 12). Thus, the pooled results of this meta-analysis were relatively robust, despite existing significant heterogeneity among most of the outcome measures.

#### Publication bias

**Figure 13:**
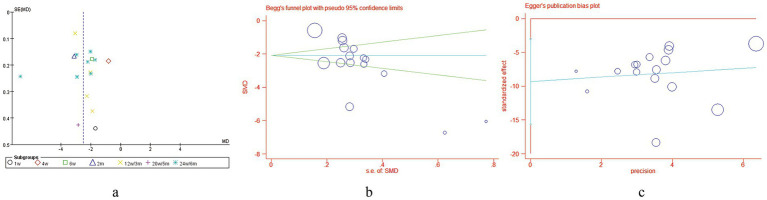
Publication bias assessment of VSS scores: **(a)** Funnel plot, **(b)** Begg’s test, **(c)** Egger’s test.

**Figure 14:**
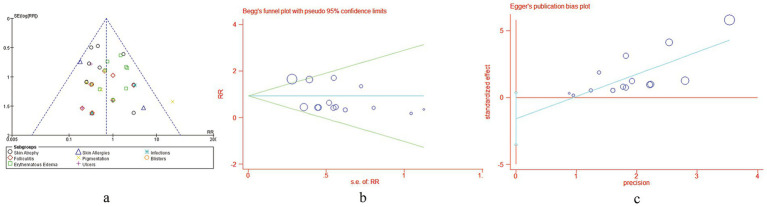
Publication bias assessment of adverse events: **(a)** Funnel plot, **(b)** Begg’s test, **(c)** Egger’s test.

The number of included studies exceeded 10 for only two outcome measures: VSS scores and adverse events. We assessed publication bias in the two meta-analyses. Visual inspection of the funnel plot revealed asymmetry for VSS scores, and subsequent quantitative analyses confirmed significant publication bias (Begg’s test: z = 2.39, *p* = 0.017; Egger’s test: t = −3.16, *p* = 0.007) (Figure 13). However, visual inspection of the funnel plot for adverse event incidence showed symmetry, suggesting no significant publication bias. This was further supported by statistical tests (Begg’s test: z = 0.44, *p* = 0.622; Egger’s test: t = −1.76, *p* = 0.103) (Figure 14).

#### Level of evidence

GRADE evidence quality evaluation was conducted on nine outcomes, encompassing VSS scores, pruritus scores, pain scores, scar thickness, the level of serum TGF-*β*1, the level of serum VEGF, the level of serum EGF, the level of serum TNF-*α*, and adverse events (Table 3).

**Table 3:** Summary of GRADE on the outcomes of the efficacy of fractional CO_2_ laser therapy combined with triamcinolone acetonide injection for hypertrophic scar.

Outcome indicators	№ of studies	Study design	Risk of bias	Inconsistency	Indirectness	Imprecision	Other considerations	№ of patients		Relative (95% CI)	Absolute (95% CI)	Certainty	Importance
								EG	CG				
VSS scores	16	Randomized trials	Not serious	Serious	Not serious	Not serious	Publication bias	758	755	–	MD 2.52 lower (3.07 lower to 1.98 lower)	⨁⨁◯◯ Low	Important
Pruritus scores	5	Randomized trials	Not serious	Not serious	Not serious	Not serious	None	279	279	–	MD 0.86 lower (0.94 lower to 0.78 lower)	⨁⨁⨁⨁ High	Important
Pain scores	5	Randomized trials	Not serious	Serious	Not serious	Not serious	None	208	208	–	MD 1.04 lower (1.38 lower to 0.71 lower)	⨁⨁⨁◯ Moderate	Important
Scar thickness	8	Randomized trials	Not serious	Serious	Not serious	Not serious	None	273	242	–	MD 2.58 lower (3.36 lower to 1.81 lower)	⨁⨁⨁◯ Moderate	Important
The level of serum TGF-β1	6	Randomized trials	Not serious	Serious	Not serious	Not serious	None	286	286	–	MD 2.09 lower (2.71 lower to 1.46 lower)	⨁⨁⨁◯ Moderate	Important
The level of serum VEGF	5	Randomized trials	Not serious	Serious	Not serious	Not serious	None	251	251	–	MD 2.03 lower (2.9 lower to 1.17 lower)	⨁⨁⨁◯ Moderate	Important
The level of serum EGF	2	Randomized trials	Not serious	Not serious	Not serious	Serious	None	136	136	–	MD 17.38 lower (20.94 lower to 13.82 lower)	⨁⨁⨁◯ Moderate	Important
The level of serum TNF-α	3	Randomized trials	Not serious	Serious	Not serious	Serious	None	172	172	–	MD 1.81 lower (2.77 lower to 0.85 lower)	⨁⨁◯◯ Low	Important
Adverse events	14	Randomized trials	Not serious	Not Serious	Not serious	Not serious	None	88/2424 (3.6%)	128/2419 (5.3%)	RR 0.7 (0.54 to 0.90)	16 fewer per 1,000 (from 24 fewer to 5 fewer)	⨁⨁⨁⨁ High	Important

EG, experimental group; CG, control group; CI, confidence interval; MD, mean difference; RR, risk ratio.

## Discussion

The results of our study suggested that, compared to TA injection alone, fractional CO_2_ laser therapy combined with TA injection in the treatment of hypertrophic scars significantly reduced VSS scores, pruritus scores, pain scores, and scar thickness. Meanwhile, the combination therapy group demonstrated greater reductions in serum levels of TGF-β, VEGF, EGF, and TNF-α. Safety analysis revealed that combination therapy could reduce the incidence of skin atrophy, with no significant difference in adverse events between the two groups (such as folliculitis, erythematous edema, skin allergies, pigmentation, ulcers, infections, and blisters), suggesting a favorable safety profile.

Hypertrophic scars result from aberrant wound healing processes (33). The wound healing process comprises four distinct physiological stages: hemostasis (blood clotting), inflammation, proliferation, and tissue remodeling. Under normal conditions, wound repair proceeds without inducing hypertrophic scar formation. However, persistent inflammation or infection can disrupt this process, prolonging healing and promoting HS development (34). Pathologically, HS are characterized by dermal hyperproliferation, driven by abnormal fibroblast activity and excessive deposition of extracellular matrix (ECM) proteins-primarily collagen-along with sustained inflammation and fibrotic remodeling (manifested as connective tissue thickening and scarring). Although the molecular mechanisms underlying pathological scar formation remain unclear, current evidence implicates dysregulated myofibroblast activity and the formation of a thick, hypervascular dermis dominated by immature collagen (35).

There are various available treatment options for visible hypertrophic scars. These options include pressure therapy, silicone gel, polyurethane dressing, lasers and other light treatments, cryotherapy, surgical excision, and corticosteroid injections (36). Among them, intralesional corticosteroid injections are recommended by the International Advisory Panel on Scar Management for hypertrophic scars. Evidence indicates their high efficacy in scar regression, mediated through multiple mechanisms (37).

Triamcinolone acetonide, a corticosteroid, has been the most commonly used since the 1960s (38). It mediates therapeutic effects through four primary mechanisms: inhibition of leukocyte and monocyte migration and phagocytosis (anti-inflammatory), suppression of keratinocyte and fibroblast proliferation (antimitotic), induction of vasoconstriction to reduce tissue perfusion, and enhancement of collagen degradation (39, 40). While TA injection has demonstrated well-established clinical efficacy, its use as monotherapy faces several challenges. The dense, poorly permeable scar tissue hinders drug penetration, while suboptimal injection depth (either too shallow or too deep) reduces therapeutic efficacy. Patients may experience obvious pain during injection, and excessive drug concentration can cause hypopigmentation and telangiectasia.

Of note, our study demonstrated that the combined therapy of fractional CO_2_ laser and TA injection yielded significantly better outcomes than TA monotherapy. Fractional CO_2_ laser has emerged as an effective energy-based modality for hypertrophic scar management. Its mechanism relies on fractional photothermolysis, which vaporizes water molecules within scar tissue, disrupts fibrin structures, and generates micro-vaporization pores surrounded by thermal damage zones. This process induces thermal peeling, coagulation, and related thermal effects, ultimately forming microscopic thermal zones (MTZs) (41). Studies have shown that fractional CO_2_ laser therapy promotes apoptosis, inhibits fibroblast proliferation, modulates angiogenesis, and regulates cytokine concentrations in scar tissue. Furthermore, it alters collagen composition (particularly the type I/III collagen ratio), thereby enhancing tissue remodeling. These synergistic mechanisms collectively improve HS (42, 43).

The improvement of hypertrophic scar condition can be assessed through various indicators, including clinical observations such as reductions in scar height and thickness, enhanced flexibility, and alleviation of itching, as well as histopathological and immunohistochemical evaluations, along with the expression of inflammatory mediators involved in the pathogenesis (41). Currently, numerous tools are available to assess the appearance of hypertrophic scars, among which the VSS is the most widely used. In this study, the 95% CI for the reduction in VSS scores in the combination therapy group ranged from 1.98 to 3.07, with the minimum clinical difference being 1.98. We attempted to explain this by identifying the minimal clinically important difference for the VSS, but without success. In the study conducted by Mahar PD et al. (44), which evaluated the therapeutic efficacy of fractional ablative CO₂ lasers for hypertrophic scars based on VSS scores, the overall reduction in total VSS was 2.15—our minimal clinically important difference of 1.98 was very close to this value. In addition, since the VSS is a 13-point scoring system composed of four subscales (pigmentation, vascularity, pliability, and scar height), each subscale has a scoring range of 0–3 or 0–4 points. An average improvement of approximately 15% (1.98/13) across all dimensions—corresponding to a score reduction of 0.45 to 0.6 points—can be intuitively perceived by both patients and physicians. Therefore, the reported improvement in effect size is not only statistically significant but also represents a marked therapeutic effect with clear clinical value, which can be used to guide treatment decisions. Notably, very high statistical heterogeneity (I^2^ often >90%) and confirmed publication bias for VSS have substantially reduced the certainty of the pooled effect estimates, even though the direction of effect is consistent. As our research results show, the combination therapy demonstrated superior efficacy compared to TA monotherapy, significantly reducing the VSS score, decreasing scar thickness, and improving pruritus and pain scores, which indicates a synergistic therapeutic effect. At the molecular level, excessive macrophages in keloid tissue promote the transformation of fibroblasts into myofibroblasts by secreting TGF-*β* (45). The overexpression of TGF-β1 enhances fibroblast proliferation and ECM deposition, thereby exacerbating scarring (46). TNF-*α*, a common pro-inflammatory factor, enhances TGF-β1 expression, thereby facilitating scar formation (47). The secretion of VEGF stimulates fibroblast proliferation and increases type I collagen synthesis (48). Excessive EGF expression disrupts the balance of cell proliferation, leading to excessive wound healing. Overexpressions of cytokines (e.g., EGF and VEGF) promote extensive neovascularization in the dermis, leading to excessive proliferation and migration of fibroblasts, as well as reduced apoptosis. This, in turn, aggravates the development of hypertrophic scars (49). Combination therapy improves hypertrophic scars through reducing the expression of the aforementioned laboratory indicators, and this aligns with its more pronounced effects in lowering VSS scores, alleviating pain and pruritus, and reducing scar thickness. In safety assessments, we were surprised to find that the combination of fractional CO₂ laser and TA injection significantly reduced the incidence of skin atrophy compared to TA monotherapy. All other reported adverse events were well-tolerated and did not interfere with treatment, further demonstrating the favorable safety profile of this combined approach. The GRADE assessment was applied to evaluate the quality of evidence. For the main outcome, weak recommendation was provided. Based on the “low” certainty for VSS, combination therapy should be applied cautiously in practice guidance; however, for additional outcomes, most recommendations were of moderate quality. Overall, clinical decision regarding the combination therapy of fractional CO_2_ laser and TA injection for hypertrophic scars should comprehensively consider each patient’s overall condition.

Our study has several limitations. First, although we searched major domestic and international databases, all the included literature originated from China, with participants exclusively Chinese. As is well known, race/ethnicity may influence the predisposition to hypertrophic scarring, which could affect the recognition and generalizability of our conclusions. Thus, this combined therapy warrants validation in diverse populations before broader implementation. Second, among the included studies, only eleven described random sequence generation methods, and no study reported the allocation concealment or blinding, and the overall methodological quality was generally suboptimal. Regarding subjective outcomes (e.g., VSS, pruritus, pain), due to the lack of allocation concealment and blinding, the interpretation of these patient-reported or observer-assessed outcomes should be undertaken with caution. This has exerted a certain degree of impact on the synthesis of the results and the confidence in the evidence. In addition, subgroup analyses did not materially reduce heterogeneity, and unmeasured variations in laser or TA protocols and follow-up duration are likely contributors. Meanwhile, most outcomes were graded as moderate or weak recommendations. Third, some outcome measures had a limited number of supporting studies, highlighting the need for more clinical reports to enable a more comprehensive analysis.

## Conclusion

Although preliminary, available evidence indicated that the combination of fractional CO₂ laser and triamcinolone acetonide injections may be effective for treating hypertrophic scars. However, the methodological limitations of the included studies, along with the fact that all 19 RCTs were conducted in China, necessitate future large-scale, rigorous trials to confirm these findings and validate their generalizability in other ethnic populations.

## Data Availability

The original contributions presented in the study are included in the article/Supplementary material, further inquiries can be directed to the corresponding authors.

## Footnotes

1
https://gradepro.org
